# The genus *Scapheremaeus* (Acari, Oribatida, Cymbaeremaeidae) in the oribatid mite fauna of New Zealand, with description of two new species

**DOI:** 10.3897/zookeys.508.10005

**Published:** 2015-06-17

**Authors:** Sergey G. Ermilov, Maria A. Minor

**Affiliations:** 1 Tyumen State University, Tyumen, Russia; 2 Institute of Agriculture & Environment, Massey University, Palmerston North, New Zealand

**Keywords:** Oribatid mites, *Scapheremaeus*, new species, new record, key, New Zealand

## Abstract

Two new species of oribatid mites of the genus *Scapheremaeus* (Oribatida, Cymbaeremaeidae), *Scapheremaeus gibbus* **sp. n.** and *Scapheremaeus luxtoni* **sp. n.**, are described from New Zealand. *Scapheremaeus gibbus* **sp. n.** is morphologically most similar to *Scapheremaeus humeratus* Balogh & Mahunka, 1967, but differs from the latter by the number of notogastral, genital and adanal setae, morphology of bothridial setae, position of adanal lyrifissures and absence of humeral processes. *Scapheremaeus luxtoni* **sp. n.** is morphologically most similar to *Scapheremaeus yamashitai* Aoki, 1970, but differs from the latter by the morphology of notogastral and rostral setae, morphology of leg solenidia φ_2_ and development of humeral processes. The species *Scapheremaeus zephyrus* Colloff, 2010 is recorded for the first time in New Zealand. An identification key to the known New Zealand species of *Scapheremaeus* is provided.

## Introduction

*Scapheremaeus* is a large genus of oribatid mites (Acari, Oribatida, Cymbaeremaeidae), which was proposed by Berlese (1910) with *Eremaeus patella* Berlese, 1886 as type species. At present, the genus comprises more than 110 species and has a cosmopolitan distribution (except the Antarctic region) (Subías 2004, updated 2015; Ermilov and Anichkin 2015). The generic characters of *Scapheremaeus* are summarized by Colloff (2009). The identification keys to species from some regions and countries have been presented by Sitnikova (1975), Rios and Palacios-Vargas (1998), Balogh and Balogh (2002), Colloff (2010), Norton et al. (2010), and Ermilov and Anichkin (2015). The information about juvenile instars is summarized by Norton and Ermilov (2014), with some new data added by Ermilov et al. (2015).

During studies of oribatid mites from New Zealand, we discovered two new species of *Scapheremaeus*, *Scapheremaeus gibbus* sp. n. and *Scapheremaeus luxtoni* sp. n., and also found a known species, *Scapheremaeus zephyrus* Colloff, 2010, which was previously recorded only in Australia. The primary aim of our paper is to describe these species.

Three other species of *Scapheremaeus* are known from New Zealand (Hammer 1966): *Scapheremaeus emarginatus* Hammer, 1966, *Scapheremaeus insularis* Hammer, 1966 and *Scapheremaeus patella* (Berlese, 1886). The second aim of our paper is to provide an identification key for all known species of this genus in New Zealand.

## Materials and methods

The collection locality and habitat for each new species are given in the “*Material examined*” sections. Additionally, two specimens (female and male) of *Scapheremaeus zephyrus* were collected from: New Zealand, South Island, Central Otago, Old Man’s Range, 45°18'58"S, 169°11'45"E, 1646 m a.s.l., in soil and debris under *Dracophyllum muscoides* cushion, 17 February 2014 (M. Minor).

Specimens were mounted in lactic acid on temporary cavity slides for measurement and illustration. The body length was measured in lateral view, from the tip of the rostrum to the posterior edge of the ventral plate. Notogastral width refers to the maximum width in dorsal aspect. Lengths of body setae were measured in lateral aspect. All body measurements are presented in micrometers. Formulas for leg setation are given in parentheses according to the sequence trochanter–femur–genu–tibia–tarsus (famulus included). Formulas for leg solenidia are given in square brackets according to the sequence genu–tibia–tarsus.

General terminology used in this paper follows that of Grandjean (summarized by Norton and Behan-Pelletier 2009; Colloff 2009).

Drawings were made with a camera lucida using a Carl Zeiss transmission light microscope “Axioskop-2 Plus”. Images were obtained with an AxioCam ICc3 camera using a Carl Zeiss transmission light microscope “Axio Lab.A1”.

## Descriptions

### Scapheremaeus gibbus sp. n.

Taxon classificationAnimaliaOribatidaCymbaeremaeidae

http://zoobank.org/A7609D9D-BC17-4683-91C9-F76EB06C79BA

Figs 1–3 , 4–12 , 13–17 , 18–22

#### Diagnosis.

Body size: 270–307 × 131–147. Body surface areolate-reticulate. Costulae reduced, terminated by tubercles. Transcostula not developed. Rostral setae thin, directed medially. Lamellar setae minute. Bothridial setae globular. Humeral processes and circumdorsal scissure absent. Thirteen pairs of short, simple notogastral setae. Anterior tectum of ventral plate strongly developed. Palp femora with one seta. Five pairs of genital setae. Lyrifissures *iad* in transverse position. Monodactylous. Femora I and II with extremely large ventral expansions.

#### Description.

*Measurements*. Body length: 299 (holotype: female), 270–307 (seven paratypes: four females and three males); notogaster width: 147 (holotype), 143–151 (seven paratypes).

*Integument*. Body color light yellow-brownish. Body surface with areolate-reticulate sculpturing.

*Prodorsum*. Rostrum broadly rounded. Costulae reduced, terminated by tubercles, bearing lamellar setae. Transcostula absent. Rostral setae (*ro*, 10) thin, smooth, directed medially, inserted on transverse fold. Lamellar setae minute (*le*, 4), thin, straight, inserted nearer to bothridia than rostral setae. Interlamellar and exobothridial setae and their alveoli absent. Bothridial setae (*ss*, 22–24) globular, pigmented, with short stalk (6–8) and longer (16) head, having longitudinal ridges.

*Notogaster*. Normal in form, not flattened. Anterior margin slightly convex medially. Lenticulus (*len*) distinct. Humeral regions without processes. Centrodorsal zone forming longitudinal elongate hump-like structure. Circumdorsal scissure absent. Thirteen pairs of simple notogastral setae, located on small tubercles. Centro-dorsal part with four pairs of setae (*da*, *dm*, *lm*, *dp*). All lyrifissures (*im*, *ip*, *ih*, *ips*; except *ia*) well visible. Opisthonotal gland openings (*gla*) located posteriorly to *im*.

*Gnathosoma*. Subcapitulum longer than wide (53–57 × 32–36). Subcapitular setae thin, smooth; *a* and *m* (both 10) longer than *h* (6) and adoral setae (*or*_1_, *or*_2_, 4–6). Setae *a* slightly thicker than other. Palps (41–45) with setation 0–1–1–3–9(+ω). Solenidion free, not attached to eupathidium (*acm*). Chelicerae (53–57) with two simple, barbed setae; *cha* (16–18) longer than *chb* (12). Trägårdh’s organ long, tapered.

*Epimeral and lateral podosomal regions*. Anterior tectum strongly developed. Pedotecta I large, concave in dorsal view and scale-like in lateral view. Pedotecta II elongated, bifurcate distally in ventral view and broadly triangular in lateral view. Apodemes 1, 2, sejugal and 3 distinctly developed. Epimeral setal formula 3–1–2–2. Epimeral setae short (4), thin, smooth. Discidia (*dis*) roundly triangular.

*Anogenital region*. Five pairs of genital (*g*_1_–*g*_5_), one pair of aggenital (*ag*), two pairs of anal (*an*_1,_ *an*_2_) and two pairs of adanal (*ad*_1_, *ad*_2_) setae similar in length (4), thin, smooth, inserted on small tubercles. Lyrifissures *iad* in transverse position. Ovipositor elongated (68–77 × 32–36), lobes (36–41) longer than length of distal section (beyond middle fold; 32–36). Each of three lobes with four straight, smooth setae, ψ_1_ ≈ τ_1_ (20) longer than ψ_2_ ≈ τ_a_ ≈ τ_b_ ≈ τ_c_ (8–10). Coronal setae and their alveoli absent.

*Legs*. Monodactylous. Femora I and II with extremely large ventral expansions. Porose areas (*p.a*) slightly visible, oval. Formulas of leg setation and solenidia: I (0–4–2–4–16) [1–2–2], II (0–4–2–3–15) [1–1–1], III (1–2–1–3–14) [1–1–0], IV (0–2–1–3–12) [1–1–0]; homology of setae and solenidia as indicated in Table 1. Famuli (ε) short, slightly dilated distally. Solenidia simple, σ on genua IV minute. Setae *l* on tibiae I setiform, not modified.

**Figures 1–3.**
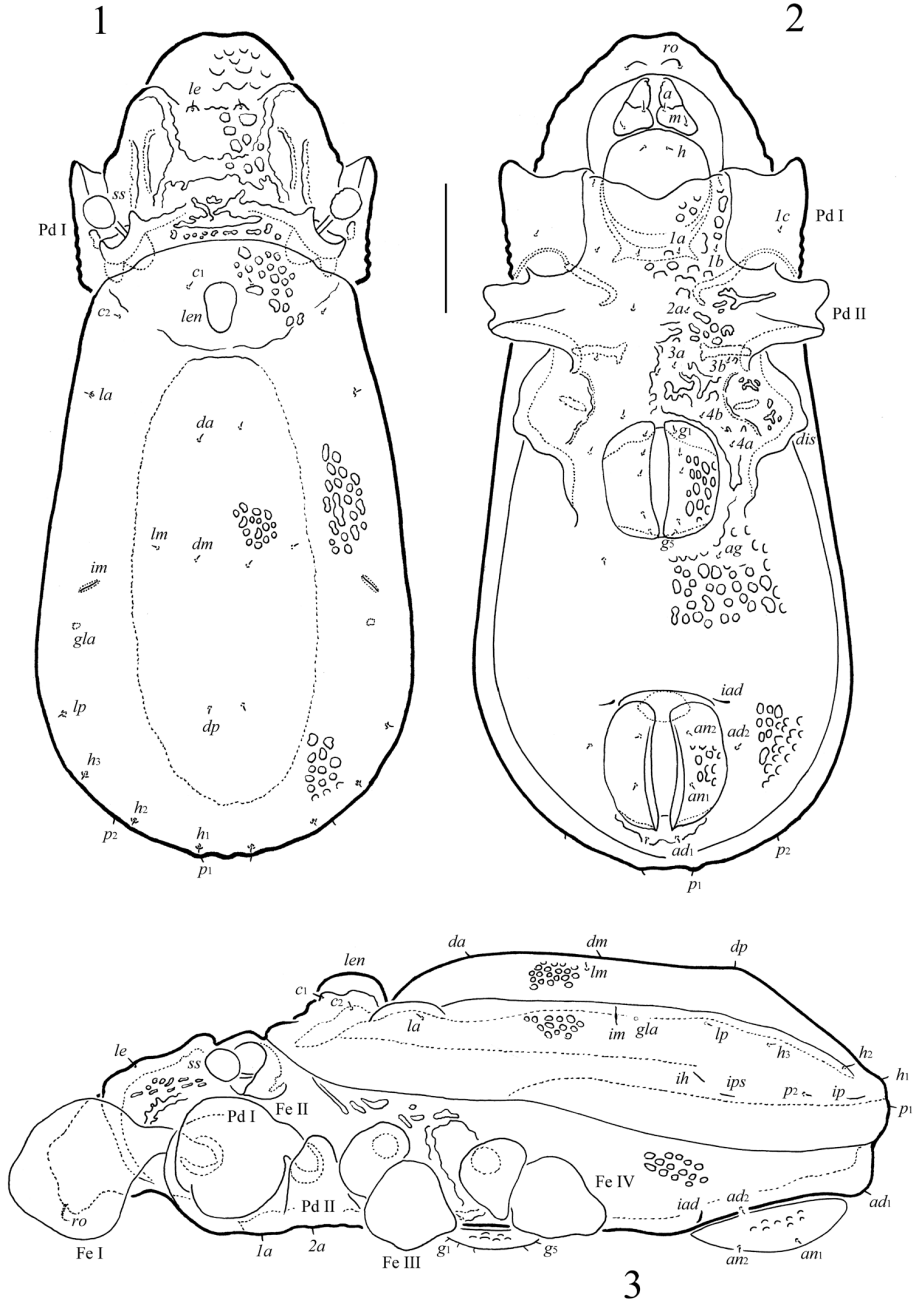
*Scapheremaeus gibbus* sp. n., adult: **1** dorsal view **2** ventral view (legs not shown) **3** lateral view (gnathosoma and legs except basal parts not shown). Scale bar 50 µm.

**Figures 4–12.**
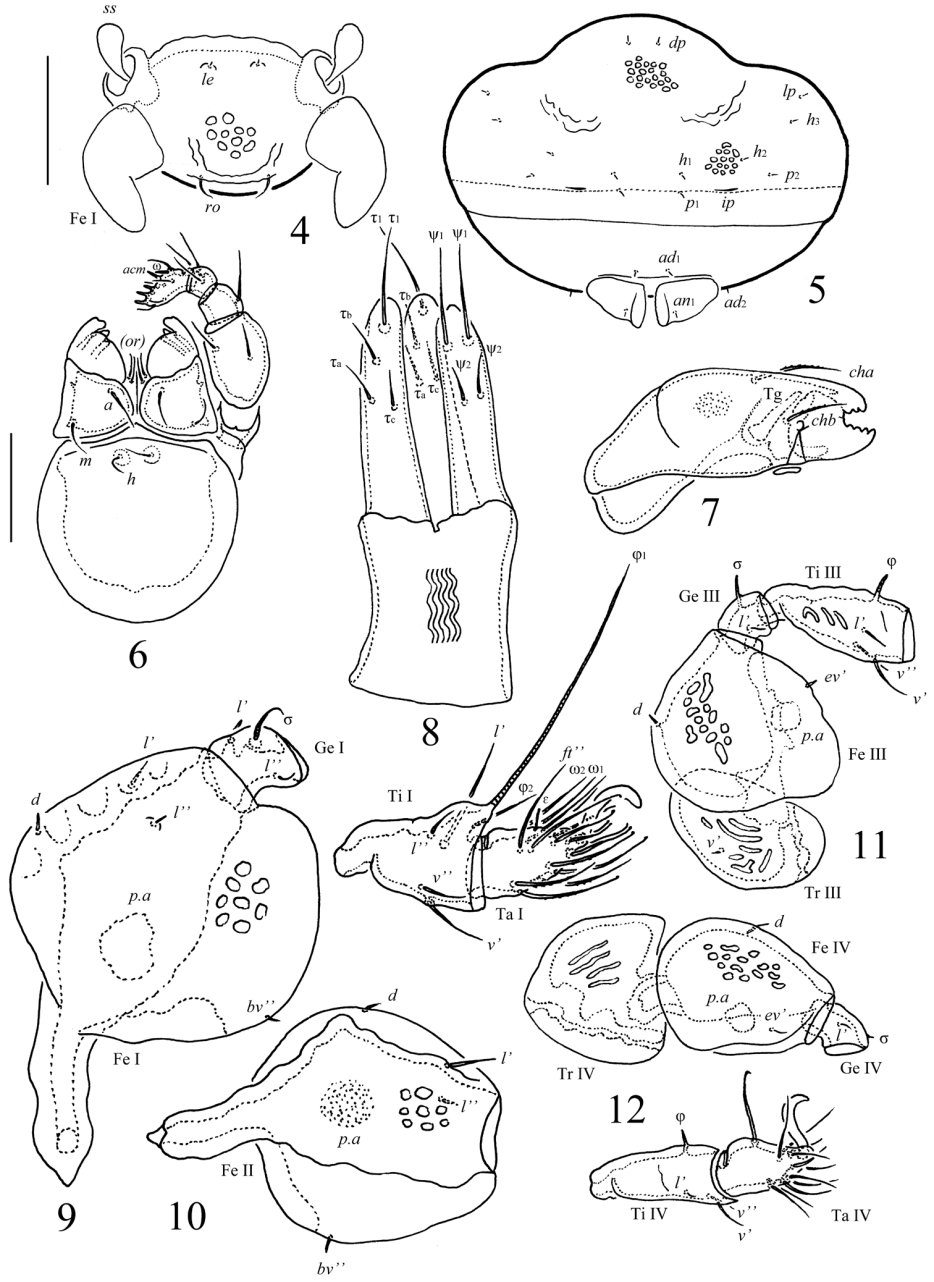
*Scapheremaeus gibbus* sp. n., adult: **4** frontal view of prodorsum (legs I except basal parts not shown) **5** posterior view **6** subcapitulum and palp **7** chelicera, antiaxial view **8** ovipositor **9** leg I, without trochanter, right, antiaxial view **10** femur of leg II, left, paraxial view **11** leg III, without tarsus, left, antiaxial view **12** leg IV, left, antiaxial view. Scale bars 50 µm (**4, 5**), 20 µm (**6–12**).

**Figures 13–17.**
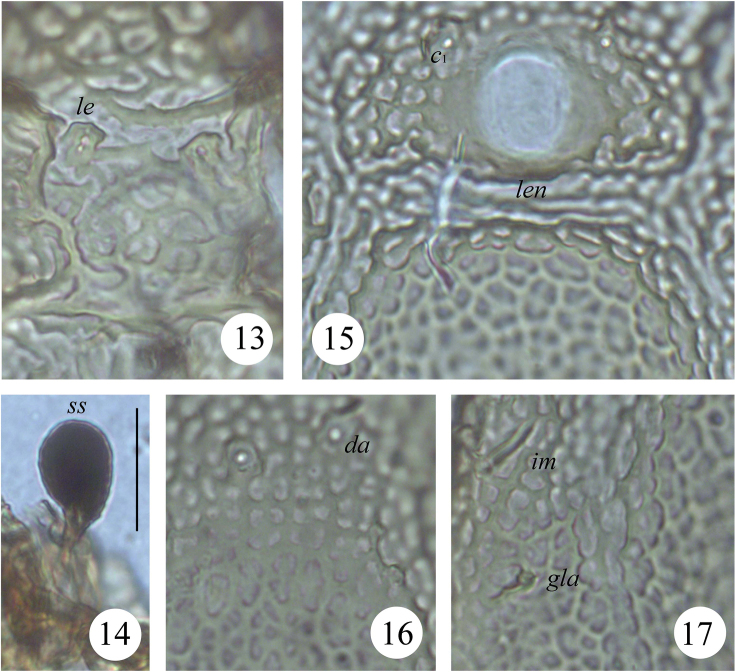
*Scapheremaeus gibbus* sp. n., dissected adult, microscope images: **13** lamellar setae and ornamentation in centro-dorsal part of prodorsum **14** bothridial seta **15** lenticulus and sculpture on anterior part of notogaster **16** sculpture on centro-dorsal part of notogaster **17** sculpturing in dorso-lateral part of notogaster. Scale bar 20 µm.

**Figures 18–22.**
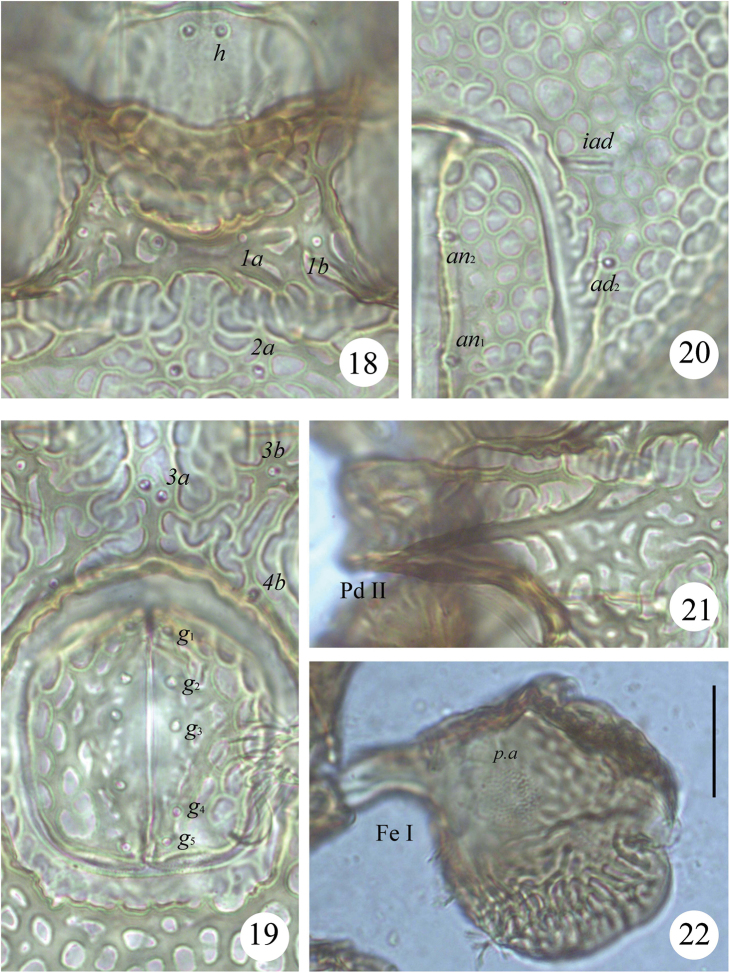
*Scapheremaeus gibbus* sp. n., dissected adult, microscope images: **18** sculpturing in anterior part of epimeral region **19** genital plates **20** left anal plate and sculpturing in adanal part of ventral plate **21** pedotecta II **22** femur I, left, paraxial view. Scale bar 20 µm.

#### Material examined.

Holotype (female) and seven paratypes (four females and three males): New Zealand, South Island, Central Otago, Old Man’s Range, 45°18'58"S, 169°11'45"E, 1646 m a.s.l., in soil and debris under *Dracophyllum muscoides* cushion, 17 February 2014, collected by M. Minor.

#### Type deposition.

The holotype and two paratypes are deposited in the New Zealand National Arthropod Collection, Auckland, New Zealand; two paratypes are deposited in the collection of the Senckenberg Institution, Frankfurt, Germany; three paratypes are deposited in the collection of the Tyumen State University Museum of Zoology, Tyumen, Russia.

#### Etymology.

The specific name *gibbus* refers to the clearly convex centrodorsal notogastral region, forming longitudinal elongate hump-like structure.

#### Remarks.

The new species is most similar to *Scapheremaeus humeratus* Balogh & Mahunka, 1967 from Congo (see Balogh and Mahunka 1967) in having small body size, monodactylous legs, simple notogastral setae, areolate body surface, and absence of circumdorsal furrow. However, it differs from the latter by the presence of 13 pairs of notogastral setae (versus 11), globular bothridial setae (versus fusiform), five pairs of genital setae (versus six), two pairs of adanal setae (versus three), transverse position of adanal lyrifissures (versus longitudinal) and absence of humeral processes (versus well developed).

### Scapheremaeus luxtoni sp. n.

Taxon classificationAnimaliaOribatidaCymbaeremaeidae

http://zoobank.org/8F13864C-3F7C-44A9-B831-2C4892BB7F89

Figs 23–25 , 26–35 , 36–42 , 43–47

#### Diagnosis.

Body size: 381–415 × 199–232. Centro-dorsal part of notogaster areolate. Dorso-lateral parts of notogaster and ventral plate tuberculate. Costulae and transcostula strong. Rostral setae thin, straight. Lamellar setae minute. Bothridial setae globular. Humeral processes small, rounded. Circumdorsal scissure present. Ten pairs of short, simple notogastral setae. Palp femora with two setae. Six pairs of genital setae. Lyrifissures *iad* longitudinally oriented. Tridactylous.

#### Description.

*Measurements*. Body length: 381 (holotype: male), 381–415 (three paratypes: all females); notogaster width: 199 (holotype), 232 (same for three paratypes).

*Integument*. Body color light yellow-brownish. Anterior part of prodorsum and centro-dorsal part of notogaster with areolate sculpturing. Dorso-lateral parts of notogaster and ventral plate with elongated ridge-like tubercles.

*Prodorsum*. Rostrum broadly rounded. Costulae (*cos*) distinct, forming slightly visible *X*-structure, terminated by large tubercles, which connected by thick transcostula (*tcos*). Rostral setae (6) thin, straight, inserted on transverse fold. Lamellar setae (4) minute, inserted nearer to rostral setae than to bothridia. Interlamellar and exobothridial setae and their alveoli absent. Bothridial setae (22–24) globular, pigmented, with short stalk (6) and longer (16–18) head, having longitudinal ridges.

*Notogaster* flattened. Anterior margin straight. Lenticulus distinct. Humeral processes (*Hp*) slightly developed, tubercle-like in dorsal view and rounded in lateral view. Centrodorsal zone with longitudinal elongate hump-like structure. Circumdorsal scissure (*f*) present. Ten pairs of simple notogastral setae, located on small tubercles. Centro-dorsal part with two pairs of setae (*lm*, *lm*), both inserted near to scissure. All lyrifissures (except *ia*) well visible. Opisthonotal gland openings located medially to *ih*.

*Gnathosoma*. Subcapitulum longer than wide (82–90 × 61–69). Subcapitular setae thin, smooth; *a* and adoral setae (all 10) longer than *m* and *h* (both 6–8). Setae *a* slightly thicker than other. Palps (53–61) with setation 0–2–1–3–9(+ω). Solenidion free, not attached to eupathidium. Chelicerae (82–90) with two simple, barbed setae (both 16–20). Trägårdh’s organ long, tapered.

*Epimeral and lateral podosomal regions*. Anterior tectum slightly developed. Pedotecta I of medium size, concave in dorsal view and scale-like in lateral view. Pedotecta II elongated, bifurcate distally in ventral view and broadly triangular in lateral view. Apodemes 1, 2, sejugal and 3 distinctly developed. Epimeral setal formula 3–1–2–2. Epimeral setae short (4), thin, smooth. Discidia roundly triangular.

*Anogenital region*. Six pairs of genital, one pair of aggenital, two pairs of anal and three pairs of adanal setae similar in length (4), thin, smooth, inserted on small tubercles. Lyrifissures *iad* longitudinally oriented. Ovipositor elongated (52–56 × 41–45), lobes (32–36) longer than length of distal section (beyond middle fold; 20). Each of three lobes with four straight, smooth setae, ψ_1_ ≈ τ_1_ (24–28) longer than ψ_2_ ≈ τ_a_ ≈ τ_b_ ≈ τ_c_ (16). Coronal setae and their alveoli absent.

*Legs*. Tridactylous. Porose areas slightly visible, oval. Formulas of leg setation and solenidia as in *Scapheremaeus gibbus* sp. n.; homology of setae and solenidia as indicated in Table 1. Famuli short, slightly dilated distally. Solenidia (except simple ω on tarsi and φ_1_, and thin σ on genua I) dilated distally. Setae *l* on tibiae I setiform, not modified.

**Figures 23–25.**
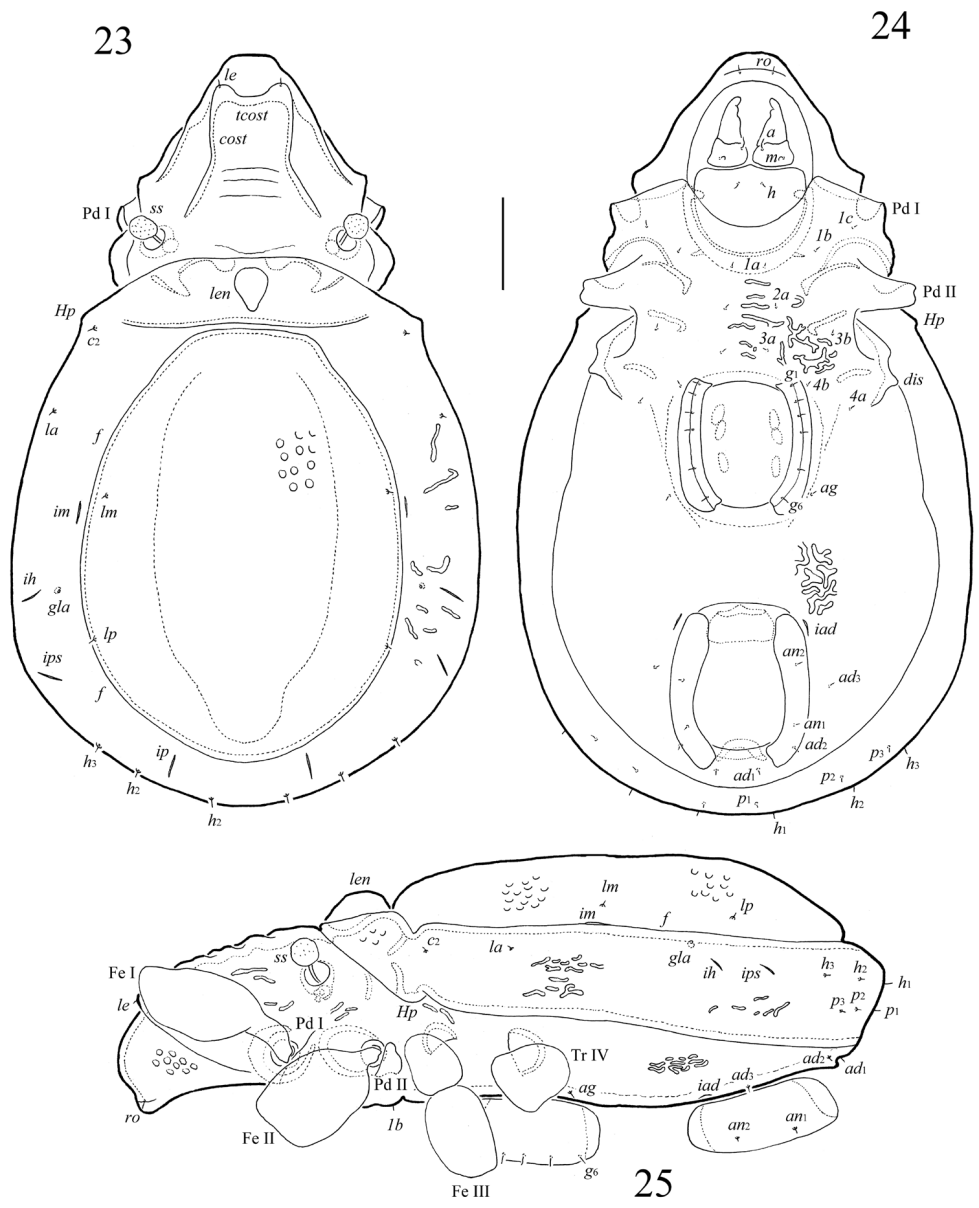
*Scapheremaeus luxtoni* sp. n., adult: **23** dorsal view **24** ventral view (legs not shown) **25** lateral view (gnathosoma and legs except basal parts not shown). Scale bar 50 µm.

**Figures 26–35.**
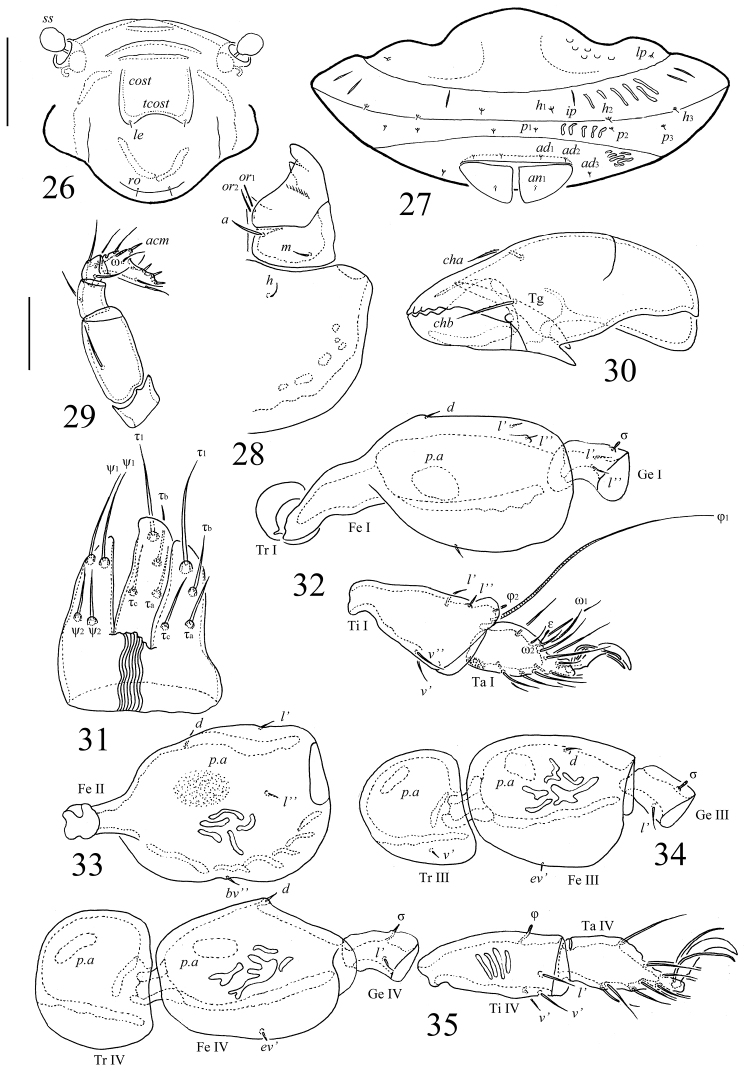
*Scapheremaeus luxtoni* sp. n., adult: **26** frontal view of prodorsum **27** posterior view **28** subcapitulum **29** palp **30** chelicera, antiaxial view **31** ovipositor **32** leg I, right, antiaxial view **33** femur of leg II, left, paraxial view **34** trochanter, femur and genu of leg III, left, antiaxial view **35** leg IV, left, antiaxial view. Scale bars 50 µm (**26, 27**), 20 µm (**28–35**).

**Figures 36–42.**
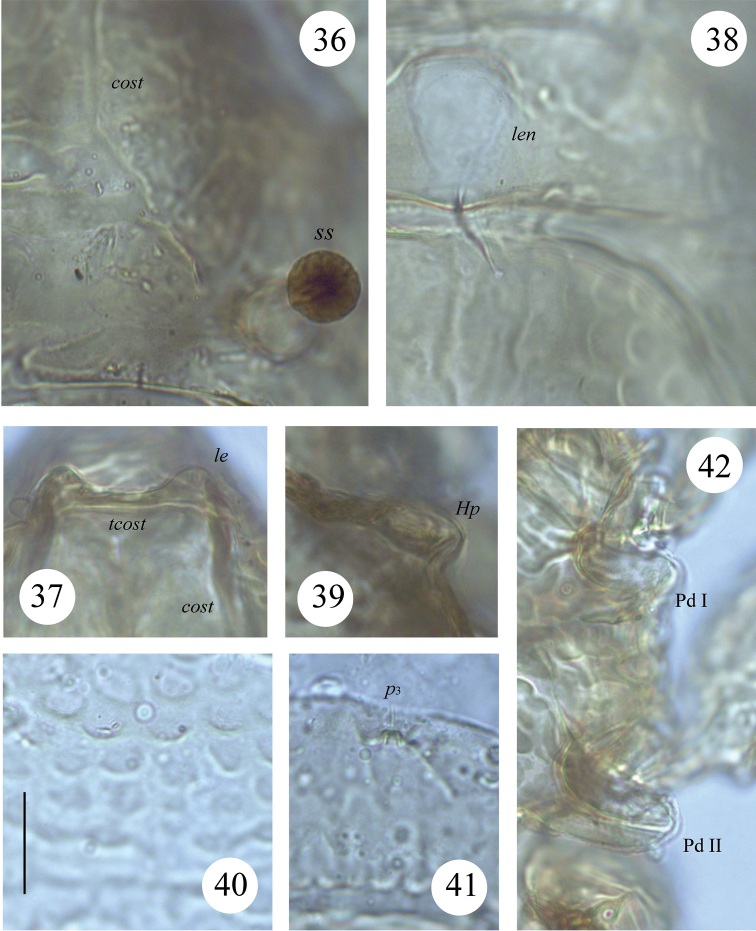
*Scapheremaeus luxtoni* sp. n., dissected adult, microscope images: **36** bothridial seta and sculpture of latero-basal part of prodorsum **37** costulae and transcostula **38** lenticulus and sculpture on latero-anterior part of notogaster **39** humeral process, right, dorsal view **40** sculpture on centro-dorsal part of notogaster **41** notogastral seta *p*_3_ **42** pedotecta I and II. Scale bar 20 µm.

**Figures 43–47.**
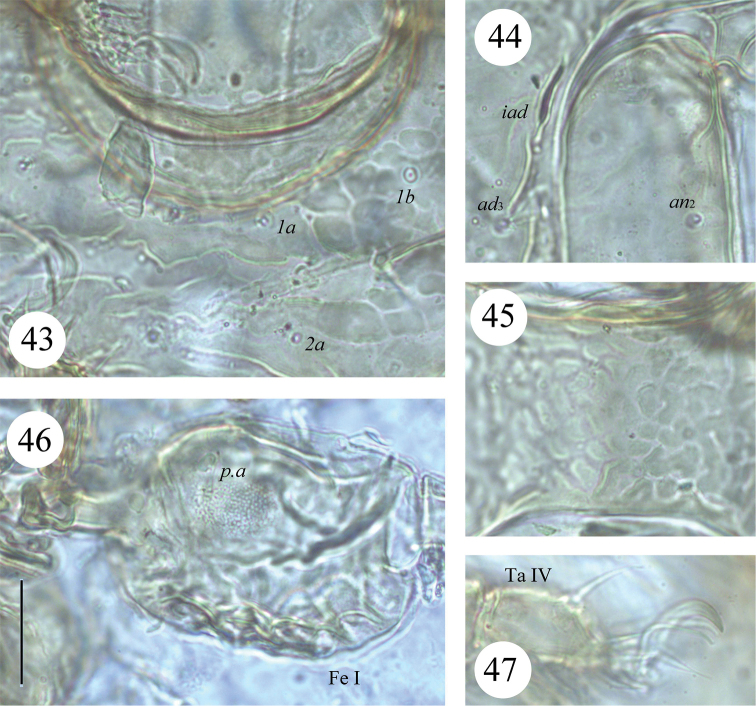
*Scapheremaeus luxtoni* sp. n., dissected adult, microscope images: **43** sculpture on anterior part of epimeral region **44** anterior part of right anal plate **45** sculpture between genital and anal apertures **46** femur I, left, paraxial view **47** tarsus IV, left, antiaxial view. Scale bar 20 µm.

#### Material examined.

Holotype (male) and three paratypes (all females): New Zealand, South Island, Central Otago, Pisa Range, 44°52'19"S, 169°10'30"E, 1880 m a.s.l., in soil and debris under *Dracophyllum muscoides* cushion and in the soil outside of *Dracophyllum muscoides* cushion, 18 February 2014, collected by M. Minor.

#### Type deposition.

The holotype and one paratype are deposited in the New Zealand National Arthropod Collection, Auckland, New Zealand; one paratype is deposited in the collection of the Senckenberg Institution, Frankfurt, Germany; one paratype is deposited in the collection of the Tyumen State University Museum of Zoology, Tyumen, Russia.

#### Etymology.

The specific name is dedicated to the well-known acarologist Malcolm Luxton, for his extensive contributions to our knowledge of New Zealand oribatid mite fauna.

#### Remarks.

The new species is similar to *Scapheremaeus yamashitai* Aoki, 1970 from Japan (see Aoki 1970; Fujikawa 2002) in having circumdorsal furrow, tridactylous legs, costulae and transcostula, ten pairs of minute notogastral setae and areolate centrodorsal region of notogaster. However, it differs from the latter by the presence of thin notogastral setae (versus thickened), straight rostral setae (versus curved medially), short and dilated distally leg solenidia φ_2_ (versus long and simple) and slightly developed humeral processes (versus well developed).

Also, in having circumdorsal furrow, tridactylous legs, costulae, minute notogastral setae, straight rostral setae and areolate centrodorsal region of notogaster, *Scapheremaeus luxtoni* sp. n. is similar to *Scapheremaeus zephyrus* Colloff, 2010 from Australia (see Colloff 2010) and New Zealand (our data). However, it differs from the latter by the presence of large tubercle-like distal parts of costulae (versus small), strong transcostula (versus absent), ten pairs of thin notogastral setae (versus nine pairs and thickened) and three pairs of adanal setae (versus two pairs).

### Key to species *Scapheremaeus* from New Zealand

**Table:** 

1	Notogastral circumdorsal scissure absent; costulae reduced, represented by tubercle-like cusps; legs monodactylous	**2**
–	Notogastral circumdorsal scissure present; costulae well developed; legs tridactylous	**3**
2	Notogaster with 13 pairs of setae; notogastral setae simple; leg femora I, II with extremely large ventral expansions; body size: 270–307 × 131–147	***Scapheremaeus gibbus* sp. n.**
–	Notogaster with 10 pairs of setae; notogastral setae dilated distally; leg femora I, II without extremely large expansions; body length: 330	***Scapheremaeus emarginatus* Hammer, 1966**
3	Costular cusps elongate conical; notogastral setae dilated distally; body length: 420	***Scapheremaeus insularis* Hammer, 1966**
–	Costular cusps tubercle-like, not elongated; notogastral setae simple or slightly thickened	**4**
4	Notogaster with 14 pairs of setae; centro-dorsal notogastral setae (*da*, *dm*, *dp*) developed; body size: 360–495 × 284	***Scapheremaeus patella*** (Berlese, 1886)^1^
–	Notogaster with 9–10 pairs of setae; centro-dorsal notogastral setae (*da*, *dm*, *dp*) not developed	**5**
5	Notogaster with 9 pairs of setae (*p*_3_ not developed); two pairs of adanal setae; transcostula absent; body size: 384–391 × 202–211	***Scapheremaeus zephyrus* Colloff, 2010**
–	Notogaster with 10 pairs of setae (*p*_3_ developed); two pairs of adanal setae; transcostula present; body size: 381–415 × 199–232	***Scapheremaeus luxtoni* sp. n.**

## Supplementary Material

XML Treatment for Scapheremaeus gibbus

XML Treatment for Scapheremaeus luxtoni

## Footnotes

1 See also redescriptions in Hammer (1966), Mahunka (1977), Mahunka and Mahunka-Papp (1995).
